# Tumor Induced Hepatic Myeloid Derived Suppressor Cells Can Cause Moderate Liver Damage

**DOI:** 10.1371/journal.pone.0112717

**Published:** 2014-11-17

**Authors:** Tobias Eggert, José Medina-Echeverz, Tamar Kapanadze, Michael J. Kruhlak, Firouzeh Korangy, Tim F. Greten

**Affiliations:** 1 Gastrointestinal Malignancy Section, Center for Cancer Research, National Cancer Institute, National Institutes of Health, Bethesda, Maryland, United States of America; 2 Department of Gastroenterology, Hepatology and Endocrinology, Hannover Medical School, Hannover, Germany; 3 Experimental Immunology Branch, Center for Cancer Research, National Cancer Institute, National Institutes of Health, Bethesda, Maryland, United States of America; Institute of Hepatology - Birkbeck, University of London, United Kingdom

## Abstract

Subcutaneous tumors induce the accumulation of myeloid derived suppressor cells (MDSC) not only in blood and spleens, but also in livers of these animals. Unexpectedly, we observed a moderate increase in serum transaminases in mice with EL4 subcutaneous tumors, which prompted us to study the relationship of hepatic MDSC accumulation and liver injury. MDSC were the predominant immune cell population expanding in livers of all subcutaneous tumor models investigated (RIL175, B16, EL4, CT26 and BNL), while liver injury was only observed in EL4 and B16 tumor-bearing mice. Elimination of hepatic MDSC in EL4 tumor-bearing mice using low dose 5-fluorouracil (5-FU) treatment reversed transaminase elevation and adoptive transfer of hepatic MDSC from B16 tumor-bearing mice caused transaminase elevation indicating a direct MDSC mediated effect. Surprisingly, hepatic MDSC from B16 tumor-bearing mice partially lost their damage-inducing potency when transferred into mice bearing non damage-inducing RIL175 tumors. Furthermore, MDSC expansion and MDSC-mediated liver injury further increased with growing tumor burden and was associated with different cytokines including GM-CSF, VEGF, interleukin-6, CCL2 and KC, depending on the tumor model used. In contrast to previous findings, which have implicated MDSC only in protection from T cell-mediated hepatitis, we show that tumor-induced hepatic MDSC themselves can cause moderate liver damage.

## Introduction

Infections, toxins, radiation, neoplasms, ischemia and trauma cause liver injury. The degree of liver injury depends on both, direct (agent dependent) and indirect (immune mediated) effects, since different cells of the innate immune system are rapidly recruited to the site of liver injury, where they aggravate liver damage [1]–[3]. On a molecular level, there are different mechanisms that can cause liver injury. For instance, detoxification of exogenous substances renders the liver susceptible to oxidative stress, which is produced during metabolism of toxic exogenous substances [4]. Acetaminophen [5] and alcohol [4] have been shown to exert a direct toxic effect through reactive oxygen species (ROS) or intermediate metabolites on hepatocytes. However, in addition to these mechanisms these agents also cause immune-mediated liver injury.

The contribution of the innate immune system to liver injury is universally acknowledged and has been extensively reviewed [6]–[10]. Not only the innate immune system in general, but more specifically the accumulation of neutrophils and macrophages can cause liver damage [8], [11]. In alcoholic liver disease, activated Kupffer cells produce TNF-α, which induces apoptosis in hepatocytes through TNF-α receptor binding [12]; thereby contributing to hepatocyte cell death and hepatic inflammation [13], [14]. This sterile cell death can trigger Kupffer cells to secrete the acute inflammatory response cytokine IL-1 [15], which can lead to recruitment of neutrophils to the liver. In acetaminophen induced liver injury, the depletion of these infiltrating neutrophils protects mice from severe hepatotoxicity [1]. These cells also play a pivotal role not only in drug-induced liver injury as described above, but also in liver damage caused by obesity, i.e. non-alcoholic steatohepatitis. In mouse models of dietary-induced non-alcoholic steatohepatitis, liver inflammation was aggravated by accumulation of immature myeloid cells or macrophages [16], [17].

Immature myeloid cells with immune suppressive ability are also termed myeloid-derived suppressor cells (MDSC). These MDSC were initially found to accumulate in tumor bearing hosts [18]. More recently, they have also been identified in trauma and chronic infections [19]. MDSC are a heterogeneous population of immature myeloid cells and comprise myeloid progenitors at different stages of the differentiation, such as precursors of granulocytes, macrophages and dendritic cells (DC). They can be found as tumor infiltrating cells, in blood, bone marrow, spleen and liver. In tumor-bearing mice, MDSC are identified by their co-expression of CD11b and Gr-1. The hallmark of MDSC is their ability to suppress both adaptive and innate immune responses through multiple mechanisms. Their accumulation in livers has been shown to protect from liver injury and to dampen T cell mediated-hepatitis [20]–[23].

Recently, our group investigated antibody-mediated hepatic MDSC depletion [24]. In addition to the finding, that anti-Gr-1 antibody failed to deplete MDSC in the liver, we observed an increase in alanine aminotransferase (ALT) and aspartate aminotransferase (AST) in EL4 subcutaneous tumor bearing mice. Therefore, we set out to study the effect of hepatic MDSC in different models of subcutaneous tumor-bearing mice in more detail. Here, we provide evidence that hepatic MDSC accumulation in tumor bearing mice can causes mild liver damage. MDSC-induced liver damage was tumor specific as not all tumor models investigated caused liver injury, although MDSC expansion was observed in all models.

## Materials and Methods

### Mice and cell lines

8–10 week-old female C57BL/6 and BALB/c were obtained from NCI/Frederick (Frederick, USA). EL4 (lymphoma), RIL175 (hepatocellular carcinoma [25]) and B16 (melanoma) tumor cell lines on C57BL/6 background and CT26 (colon carcinoma) and BNL (hepatocellular carcinoma) tumor cell lines on BALB/c background were used for subcutaneous tumor models. EL4 [26], B16 [27] and CT26 [28] cell lines were a kind gift of Dr. Drew Pardoll (The Johns Hopkins University, Baltimore, USA), The BNL cell line was generously provided by Dr. Jesus Prieto (University of Navarra, Spain; [29]) and the RIL175 cell line was obtained from Dr. Lars Zender (University Hospital of Tübingen, Germany; [25], [30]). All experiments were performed according to the institutional guidelines and approved by the National Cancer Institute Bethesda Animal Care and Use Committee (Bethesda, MD, USA).

### Animal experiments

1×10^6^ tumor cells were injected subcutaneously into the left flank of 8–10 week-old female mice. Mice were sacrificed, when subcutaneous tumors reached 15 mm or 20 mm mean diameter. ALT and AST levels were determined in mouse sera and livers were collected for immune cell analysis or fixed in 10% Formaldehyde for histology and TUNEL assays. TUNEL stainings were performed using the ApopTag Peroxidase In Situ Apoptosis Detection Kit (Millipore, Billerica, USA) according to manufacturer's instructions. Mouse testis served as control tissue. Liver histology slides stained with TUNEL were analyzed by counting TUNEL positive cells in 20 non-overlapping visual fields from individual specimens of 2 livers per group. Immunohistochemistry images were collected using a Zeiss AxioObserver Z1 microscope equipped a 10× plan-apochromat (N.A. 0.45) objective lens and a AxioCam MRc5 color CCD camera (Carl Zeiss Microscopy, llc., Thornwood, NY, USA).

MDSC depletion was achieved as described previously [31]. Briefly, mice were treated with 5-FU (50 µg/g body weight) when EL4 tumor surface was approximately 100 mm^2^. Saline treated mice served as controls.

For hepatic MDSC transfer, a single cell suspension was prepared from B16 subcutaneous tumor-bearing mouse livers by density gradient centrifugation (Percoll; Fisher Scientific, Pittsburgh, USA) and red blood cell lysis (ACK Lysis Buffer; Quality Biologicals), subsequently MACS-sorted using CD11b microbeads (Miltenyi Biotec Inc., San Diego, USA) and injected (5×10^7^ cells) intravenously into female C57BL/6 mice. Accumulation of transferred cells in livers of recipient mice was confirmed in a pilot experiment by transferring hepatic CD45.1^+^CD11b^+^ cells from tumor-bearing mice into naïve C57BL/6 (CD45.2^+^) mice and detection of CD45.1^+^CD11b^+^Gr-1^+^ cells in the recipient mouse liver via flow cytometry. Purity of MACS-sorted cells was assessed by flow cytometry. >95% of cells for transfer were CD11b^+^ and 75% were CD11b^+^Gr1^+^. Mice were sacrificed 16 h after transfer and serum ALT and AST levels were analyzed.

### Flow cytometry analysis

Single cell suspensions were prepared as described earlier [24]. Briefly, livers were homogenized, passed through a nylon mesh and liver-infiltrating cells were isolated by isotonic Percoll (Fisher Scientific, Pittsburgh, USA) centrifugation. RBCs were lysed using ACK lysis buffer (Quality Biological, Gaithersburg, USA). Cells were stained with the following mouse antibodies against: CD11b (Clone M1/70), Ly6G (1A8), Ly6C (HK1.4) CD3 (17A2), CD4 (GK1.5), CD8 (53–6.7), NK1.1 (PK136), CD19 (eBio1D3), CD11c (N418), B220 (RA3-6B2) and CD244 (eBio244F4) (all from eBioscience Inc., San Diego, USA) and Gr-1 (RB6-8C5; BioLegend, San Diego, USA). Flow cytometry was performed on BD FACS Calibur using BD CellQuest Pro software or LSRII using BD FACSDiva software (BD Biosciences, San Diego, USA). Data were analyzed using FlowJo software (Tree Star Inc., Ashland, USA). MDSC were defined as CD11b^+^Gr-1^+^, monocytic MDSC (M-MDSC) as CD11b^+^Ly6G^−^Ly6C^high^, granulocytic MDSC (PMN-MDSC) as CD11b^+^Ly6G^+^Ly6C^low^, Macrophages as CD11b^+^Gr-1^−^F4/80^+^, conventional DC as CD11c^+^CD11b^+^, plasmacytoid DC as CD11c^+^CD11b^−^B220^+^, CD4 T cells as CD3^high^CD4^+^, CD8 T cells as CD3^+^CD4^+^, NK cells as NK1.1^+^CD3^−^, NKT cells as NK1.1^+^CD3^low^ and B cells as CD19^+^CD3^−^.

### Cytokine assay

Mouse serum samples and tumor-conditioned media, derived from *in vitro* cultured tumor cell lines, were analyzed by Mouse Cytokine/Chemokine Magnetic bead panel (Millipore, Billerica, USA) according to manufacturer's instructions. Serum samples from tumor-bearing mice were normalized to naïve wild-type mice.

### Statistical analysis

Data were analyzed for statistical significance using Student's *t* test to compare two groups. When one control group was compared to multiple groups, One-way ANOVA was used. (Prism software; GraphPad); *p*<0.05 was considered to be statistically significant.

## Results

### Tumor-bearing mice suffer from mild liver damage

**Figure 1:**
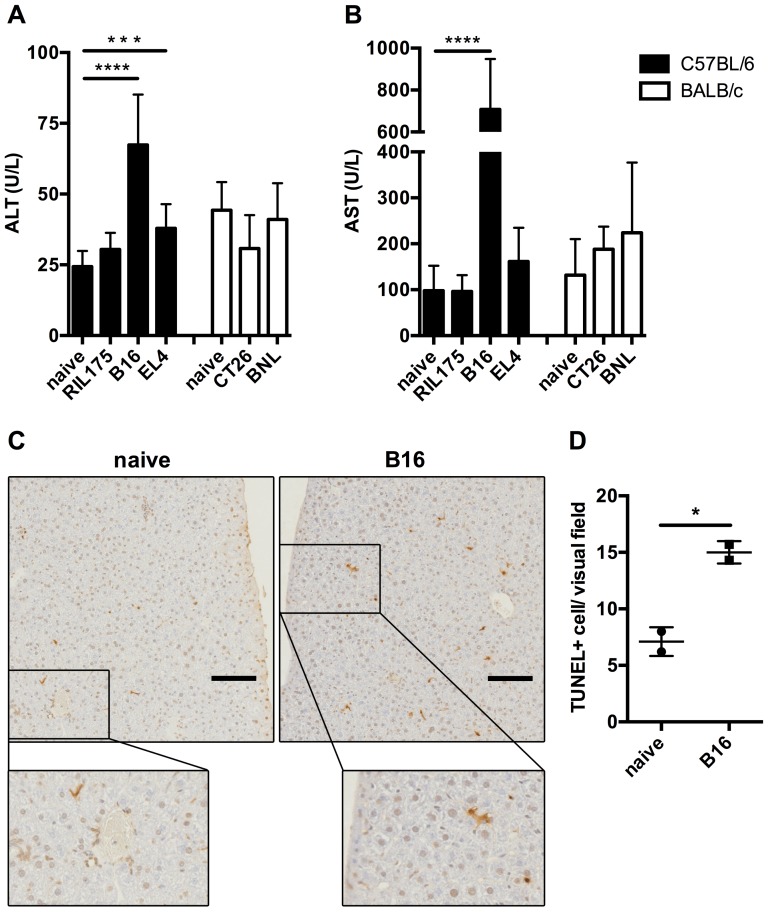
Melanoma and lymphoma subcutaneous tumor-bearing mice suffer from mild liver damage. C57BL/6 and BALB/c mice bearing indicated subcutaneous tumors were sacrificed, when tumor diameter reached 15 mm. ALT (A) and AST (B) levels were analyzed in mouse serum (N≥8 mice per tumor, N≥6 naïve mice, 3 independent experiments). Naïve C57BL/6 mice (C, left image) or mice bearing B16 subcutaneous tumors (C, right image) were sacrificed, when tumor diameter reached 20 mm. TUNEL assays were performed on liver specimen (C; scale bar  = 100 µm; N = 2 mice per group, total of 5 TUNEL assays per group) and TUNEL positive cells were counted in 20 non-overlapping visual fields. Means of TUNEL positive cells per liver section were plotted (D). C, Representative examples of visual fields are shown. Data are expressed as mean ±SEM. *p<0.05, ***p<0.001, ****p<0.0001 (by One-way ANOVA).

To investigate liver damage in subcutaneous tumor-bearing mice, we analyzed ALT and AST serum levels of BALB/c or C57BL/6 mice bearing tumors of ectodermal (B16), mesodermal (EL4) and endodermal (RIL175, BNL, CT26) origin (Figure 1A, B). B16 and EL4 tumor bearing mice had elevated levels of both liver enzymes, ALT and AST, whereas only subtle statistically not significant ALT and AST elevation were noticed in mice with other tumors. The highest increase was observed in B16 tumor-bearing mice. Both macroscopic and microscopic evaluation of livers from B16 and EL4 subcutaneous tumor-bearing mice indicated no signs for the presence of liver metastasis as a possible cause for elevated ALT and AST levels (data not shown). TUNEL assays were performed to demonstrate that the increase in ALT and AST levels in subcutaneous tumor-bearing mice was due to hepatocyte injury, i.e. apoptosis. Indeed, more apoptotic hepatocytes were seen on sections from B16 tumor-bearing mice compared to tumor-free controls (Figure 1C, D). Together, these results show that subcutaneous growth of certain tumors causes mild liver damage.

### Subcutaneous tumors induce primarily expansion of MDSC among liver immune cell subsets

**Figure 2:**
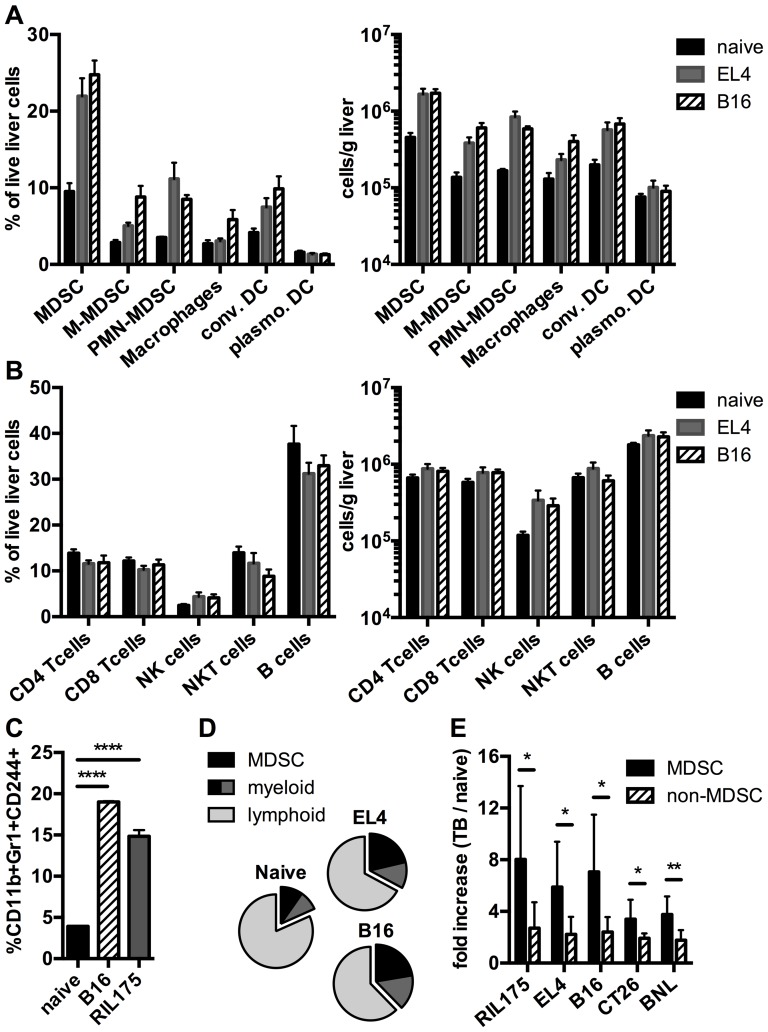
Analysis of hepatic immune cells in mice with subcutaneous tumors. C57BL/6 naïve mice or mice bearing EL4 or B16 tumors were sacrificed, when tumor diameter reached 15 mm. Hepatic immune cells were analyzed by flow cytometry and frequency and absolute cell number per gram liver were calculated for the myeloid compartment (A) and the lymphoid compartment (B) (N = 5 mice per tumor). C, Frequencies of CD11b^+^Gr-1^+^CD244^+^ cells in livers of naïve mice or mice bearing indicated tumors (N = 3 mice per group). D, Change of frequency of myeloid (including MDSC) and lymphoid cells in naïve vs. EL4 or B16 tumor-bearing mice. E, fold increase of absolute numbers of MDSC (CD11b^+^Gr-1^+^ cells) or non-MDSC (total number of liver leukocytes minus number of CD11b^+^Gr-1^+^ cells) in tumor bearing vs. naïve mice (N = 8 mice per tumor). Data are expressed as mean ±SEM. *p<0.05, **p<0.01 (C was analyzed by One-way ANOVA. E was analyzed by two-tailed Student's *t* test).

Since immune cells are capable of exacerbating liver injury, we hypothesized that the increase in ALT and/or AST in subcutaneous tumor-bearing mice is mediated by an accumulation of immune cells in the liver. To this end, we analyzed the hepatic immune subsets in mice with the highest (B16 and EL4) increase in liver enzymes in C57BL/6 mice (Figure 2). In all tumor-bearing mice the frequency and number of cells of the myeloid compartment increased compared to naïve mice (Figure 2A, B and D). Of all myeloid cells, the strongest increase was seen in MDSC. On the other hand, cells of the lymphoid compartment did not increase in frequency and only slightly increased in cell number (Figure 2B). To confirm that CD11b^+^Gr-1^+^ cells represent MDSC rather than neutrophils in our tumor-bearing mice, we studied whether CD11b^+^Gr-1^+^ cells were also positive for CD244, which has been proposed as a marker to distinguish neutrophils from granulocytic MDSC [32]. Indeed, CD11b^+^Gr1^+^ cells were also positive for CD244 in livers of B16 and RIL175 tumor-bearing mice (Figure 2C). Next, we analyzed the cell number of MDSC and non-MDSC in all tumor models used (Figure 2E). The increase of MDSC in tumor bearing vs. naïve mice was higher than the increase of non-MDSC. In summary, MDSC were the predominant immune subset expanding in livers of mice with subcutaneous tumors.

### Liver damage in subcutaneous tumor-bearing mice is MDSC mediated

**Figure 3:**
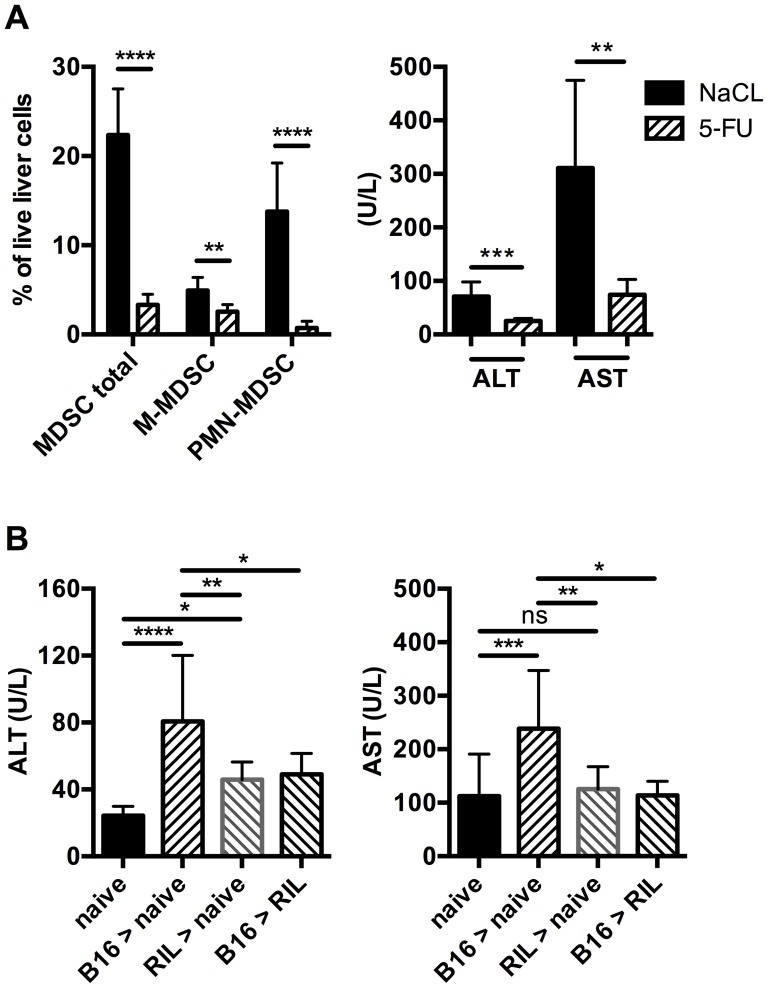
Liver injury depends on the presence of hepatic MDSC with damage-inducing potency. EL4 tumor-bearing mice were treated with 5-FU or saline. Liver immune cells were analyzed for MDSC and MDSC subsets and mouse serum was analyzed for ALT and AST levels (A) (N = 6 mice per treatment group, 2 independent experiments). B, 5×10^7^ CD11b^+^ cells isolated from livers of indicated untreated subcutaneous tumor-bearing mice were injected intravenously into naïve or RIL175 tumor-bearing recipient mice and ALT and AST serum levels were analyzed 16 h after transfer (N≥6 recipient mice, 2 independent experiments). Data are expressed as mean ±SEM. **p<0.01, ***p<0.001, ****p<0.0001 (A was analyzed by two-tailed Student's *t* test. B was analyzed by One-way ANOVA).

To determine whether the elevation of liver enzymes in our subcutaneous tumor models was MDSC mediated, we treated EL4 tumor-bearing mice with low dose 5-FU, which had been shown to deplete MDSC in tumor-bearing mice successfully [31]. As expected, the frequency of hepatic MDSC dropped significantly compared to saline treated control mice. Depletion was more prominent in the granulocytic than in the monocytic MDSC population (Figure 3A). ALT values also significantly fell; suggesting that depletion of hepatic MDSC alleviated liver damage in subcutaneous tumor bearing mice (Figure 3A). To further corroborate our result, we adoptively transferred CD11b^+^ cells from livers of liver damage-inducing B16 tumor-bearing mice into naïve C57BL/6 mice. Transferred CD11b^+^Gr-1^+^CD45.1^+^ MDSC were successfully detected in livers of recipient mice 1 and 16 h after injection, demonstrating hepatic recruitment of MDSC upon transfer (Figure S1). ALT and AST levels increased significantly 16 h after cell transfer compared to naïve mice (Figure 3B), supporting our hypothesis that hepatic MDSC were the cause of liver injury in this model.

### Subcutaneous tumors shape the potency of MDSC to cause liver damage

Since mice bearing RIL175 tumors did not have increased ALT and AST levels, we investigated the liver damage-inducing ability of MDSC from these livers by transferring hepatic CD11b^+^ cells from RIL175 tumor-bearing mice into naïve mice (Figure 3B). The recipient mice showed an ALT increase, but no increase in AST over naïve mice levels. Compared to the transfer of CD11b^+^ from B16 tumor-bearing mice, the ALT increase was lower when CD11b^+^ cell from RIL175 tumor-bearing mice were transferred, indicating less liver damage inducing potency of MDSC from RIL175 tumor-bearing mice. Furthermore, transfer of hepatic CD11b^+^ cells from B16 tumor-bearing mice into RIL175 tumor-bearing mice almost completely abolished the ALT and AST increase observed upon transfer into naïve mice (Figure 3B). Thus, MDSC partially loose their potency to cause liver damage when transferred into a host bearing a non-liver damage-inducing tumor. Together our data show, that the MDSC-inducing tumor determines the potency of MDSC to cause liver damage.

### Cytokine analysis

**Figure 4:**
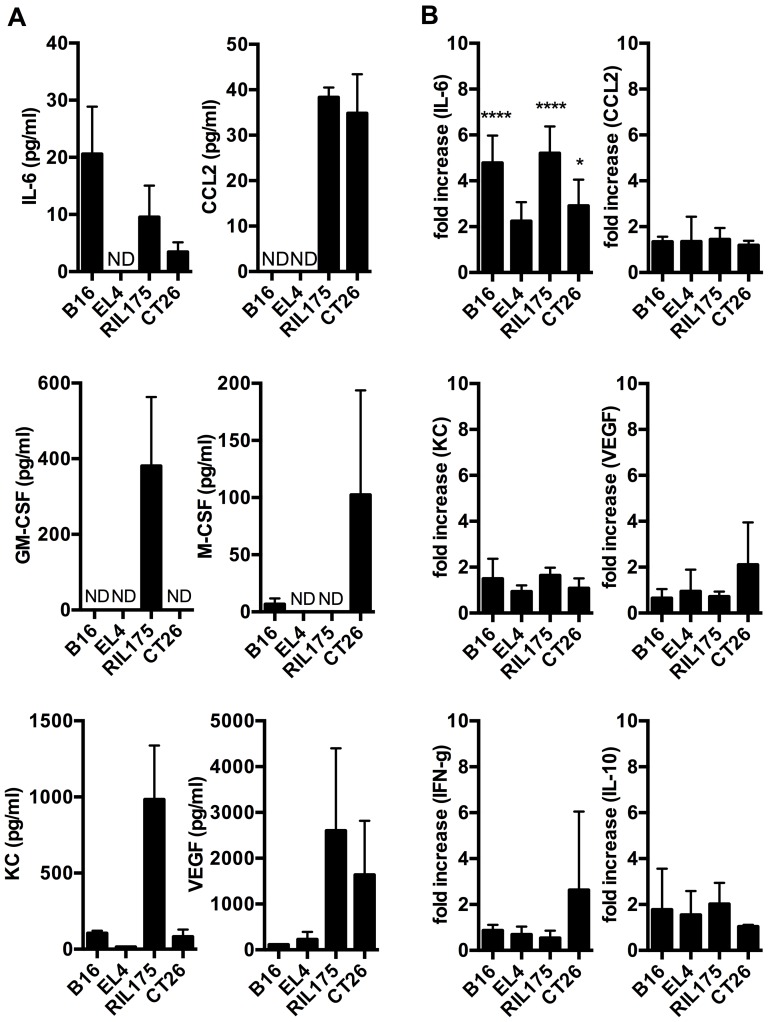
Cytokine secretion profiles of different tumor models. Duplicates of tumor-conditioned media (A, N = 4–6 media samples per tumor cell line culture) or serum samples from tumor-bearing mice (B, N = 4–6 serum samples per group) were analyzed for interleukin-6, CCL-2, GM-CSF, M-CSF, KC and VEGF (A) or interleukin-6, CCL-2, KC, VEGF, IFN-γ and interleukin 10 (B). Serum samples from tumor-bearing mice were normalized to serum from naïve wild-type mice. ND  =  not detected. Data are expressed as mean ±SEM. *p<0.05, **p<0.01, ***p<0.001 (by One-way ANOVA).

In order to determine the mechanism leading to MDSC accumulation and consecutive hepatotoxicity in tumor bearing mice with liver damage (B16 and EL4) and without (RIL175 and CT26), we next screened tumor-conditioned media (Figure 4A) and serum (Figure 4B) of tumor-bearing animals for cytokines and chemokines that have been described to expand MDSC [19] including interleukin-6, CCL-2, GM-CSF, M-CSF, KC and VEGF. The highest interleukin-6 concentration was detected in B16 tumor conditioned media, which also contained M-CSF. M-CSF was also secreted by CT26. RIL175 tumor-conditioned media contained significant amounts of a wide range of cytokines. EL4 tumor cells secreted VEGF (Figure 4A). Additionally, EL4 also secreted interleukin-4, interleukin-10 and interleukin-17 (Figure S2).

In serum of tumor-bearing mice on the other hand, interleukin-6 was elevated in all tumor models compared to tumor-free mice (Figure 4B). In contrast to our results from tumor-conditioned media, GM-CSF and M-CSF did not increase in tumor-bearing mice compared to naïve mice (data not shown). In addition to the cytokines that are known to induce MDSC accumulation, we also found an increase in serum levels of IFNγ and IL-10 in several tumor models. Serum TNF-α, interleukin-12p70, interleukin-4 and interleukin-17 remained unchanged compared to naïve mice (data not shown). In summary, each tumor cell line secreted distinct types as well as different amounts of cytokines that are known to induce MDSC accumulation. However, no increase in serum levels was found for most cytokines, which were increased in supernatants from tumor cells.

### Frequency of hepatic MDSC correlate with amount of serum transaminases

**Figure 5:**
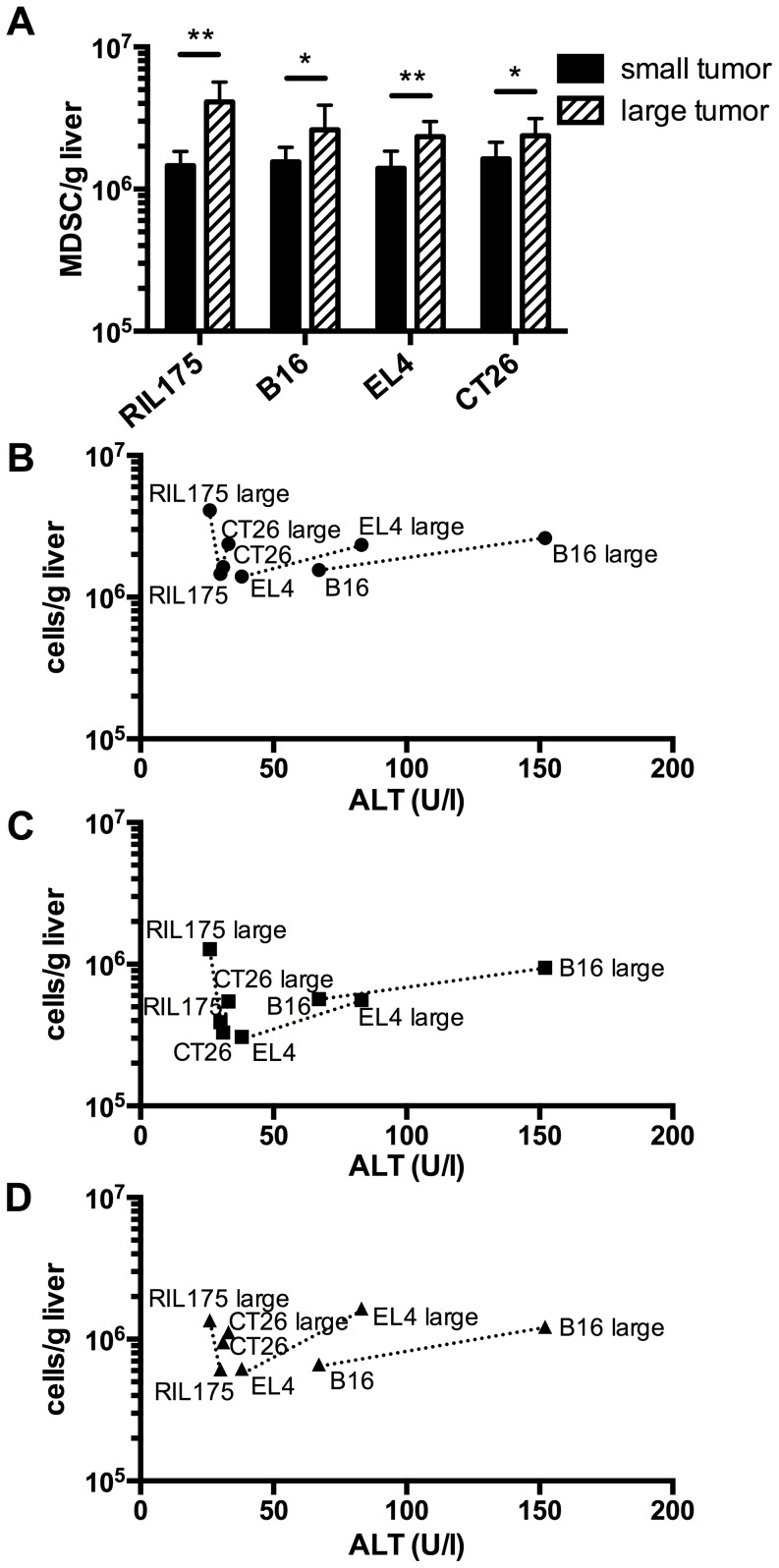
Increased expansion of liver damage-inducing MDSC exacerbates liver damage. Mice with different size subcutaneous tumors were analyzed for absolute numbers of hepatic MDSC (A and B), M-MDSC or (C), PMN-MDSC (D) and serum ALT levels (B–D). B–D, graphs correlate ALT levels with absolute numbers of MDSC and MDSC subsets. (N = 6–9 mice per tumor, 3 independent experiments). Data are expressed as mean ±SEM. *p<0.05, **p<0.01 (by two-tailed Student's *t* test).

We wondered whether increasing tumor burden in our subcutaneous model would also increase the number of hepatic MDSC and subsequently, the degree of liver damage. To this end, we analyzed hepatic MDSC numbers and liver enzymes in mice bearing tumors with two different sizes. We chose the two tumor models that induced (B16 and EL4) and two models that did not induce (RIL175 and CT26) liver damage. The number of MDSC per gram liver increased significantly in all mice bearing large tumors compared to mice bearing small tumors (Figure 5A). However, the ALT values only increased further in mice bearing liver damage-inducing B16 and EL4 tumors, indicating that a mere expansion of MDSC per se does not suffice to cause or aggravate liver damage (Figure 5B–C). Hence, these data confirmed our previous finding, that the MDSC potency to cause liver damage varied between tumor cell lines and was tumor specific. Furthermore, continued expansion of liver damage-inducing MDSC aggravated liver injury. Since total MDSC as well as the granulocytic and monocytic subset expanded similarly, we could not attribute the MDSC mediated liver damage to a specific subset.

## Discussion

Accumulation of MDSC in blood and secondary lymphoid organs of tumor-bearing mice, in which MDSC co-express CD11b and Gr-1, and cancer patients has long been recognized. The finding that CD11b^+^Gr-1^+^ cells also accumulate in disease-free livers of subcutaneous tumor-bearing mice is relatively new [33] and has also been confirmed in mice with intra-abdominal tumors [34]. More recently our group has shown, that hepatic CD11b^+^Gr-1^+^ cells in subcutaneous tumor-bearing mice actually do suppress T cell proliferation; hence, they represent MDSC [25]. In our present study, we show, that MDSC not only accumulate, but rather constitute the predominant expanding cell population in livers of subcutaneous tumor-bearing mice and that these MDSC can cause tumor-dependent mild liver damage. Furthermore, we show a correlation between liver damage-inducing hepatic MDSC numbers and severity of liver injury.

Immune cells, more specifically myeloid cells, are known to be involved in exacerbating liver injury caused by drugs, toxins, alcohol, and obesity. The degree of liver damage in these settings is aggravated by myeloid cells that are attracted to the liver through cytokines, secreted in response to hepatocyte cell death [1]–[3], [16], [17]. However, in subcutaneous tumor-bearing mice, myeloid cells accumulated in livers without initial hepatocyte insult. Among these myeloid cells, primarily MDSC accumulated and their expansion was significantly greater than the expansion of all other immune cells. Furthermore, in our melanoma and lymphoma models, hepatic MDSC triggered liver injury and the degree of liver injury increased with further expansion of these MDSC.

We established a causal link between MDSC accumulation and liver damage by depleting or transferring MDSC. Administration of anti-Gr-1 antibody is a common and widely used approach to deplete MDSC in blood and spleens of tumor-bearing mice [1], [2], [35], [36]. However, anti-Gr-1 antibody depletion does not successfully eliminate MDSC in the liver, because MDSC repopulate the liver immediately after treatment [24]. On the other hand, 5-FU treatment has been shown to selectively deplete MDSC in EL4 tumor-bearing mice [31] and was indeed successful to deplete hepatic MDSC in this study. It is noteworthy however, that treatment with 5-FU also decreases tumor sizes, which is attributed to CD8^+^ T cell activation through loss of immunosuppressive MDSC [31]. Consequently, hepatic immune cell numbers and frequencies might change. Moreover, a hypothetical direct liver damaging effect of tumor-released molecules could have been reduced with shrinking tumors and potentially could have led to the misinterpretation, that MDSC depletion alone alleviated liver damage. Indeed, cytokines might also cause hepatocyte death and liver injury directly, without harnessing immune cells as effector cells. TNF-α can bind to its receptor on hepatocytes and initiate apoptosis through pathways including ROS production and caspase-8 activation [12], [14], [37]. In our study however, TNF-α was not secreted by any of the tumor models investigated. We cannot rule out, that other tumor-secreted cytokines had a direct effect on hepatocytes, but with the data presented here, it is rather unlikely that this could have been a major contributor of liver damage, because our transfer experiments in conjunction with the depletion experiments established a direct link between MDSC and liver damage.

Production of ROS is believed to be the main mechanism by which infiltrating myeloid cells cause liver damage in settings with initial hepatic insult [7], [8], [11], [38]. Since MDSC also produce ROS [39], this mechanism could be responsible for the MDSC-mediated liver damage in our study, where an initial hepatic insult was absent. Among MDSC subsets, PMN-MDSC are the predominant subset and produce more ROS than their monocytic counterpart [40]. Accordingly, in mice with growing tumor burden and increasing ALT levels, we saw an expansion of this MDSC subset. Nevertheless, M-MDSC expanded as well, suggesting that this subtype might also contribute to MDSC-mediated liver damage. MDSC not only produce ROS, but are also known to produce a plethora of other immune suppressive factors, e.g. transforming growth factor-β (TGF-β) [18], [41]. However, TGF-β has also been recognized to induce apoptosis in hepatocytes [42]–[44] and macrophage-derived TGF-β has been shown to cause hepatocellular injury [45], providing another potential mechanism by which MDSC might cause liver damage. In summary, MDSC are equipped with means that have the potential to cause hepatocyte injury.

Several cytokines and chemokines like IL-6, CCL2, GM-CSF, M-CSF, KC and VEGF have been implicated in MDSC expansion and migration [20], [25], [46]–[51]. In our study, every tumor cell line secreted at least one of the aforementioned factors and IL-6 elevation could also be detected in the serum of tumor-bearing mice compared to tumor free controls. The combination and secreted amount of these factors varied between all cell lines; therefore, each cell line possessed an individual cytokine secretion profile. Still, each individual cytokine profile was capable of inducing hepatic MDSC expansion. Nevertheless, it is important to distinguish between mechanisms of MDSC expansion and MDSC activation, as factors that induce MDSC accumulation do not necessarily confer functional activity [19]. Cytokines whose signaling pathways converge on the transcription factor STAT3 have been reported to be the key mechanism of MDSC expansion [52], [53], while STAT1 and STAT6 signaling has been shown to be important for MDSC activity [54]–[56]. Moreover, it has been shown that the combination of GM-CSF with either G-CSF or interleukin-6 gave rise to a more immunosuppressive phenotype of MDSC than each cytokine alone, indicating that a secretion pattern of different cytokines rather than one specific cytokine is important for the function and activity of these cells [57]. Indeed, our transfer experiments showed, that the liver damage-inducing potency of MDSC was tumor-specific and our cytokine analysis revealed, that each tumor had an individual cytokine secretion profile, suggesting that these cytokine profiles determined the liver damage-inducing potency. In summary, all tumor-specific cytokine profiles in our study were capable of expanding hepatic MDSC, yet with differing potencies to cause liver damage. However, we could not establish a correlation between the accumulation of liver damage-inducing MDSC and a specific cytokine. Future experiments should dissect the role of candidate cytokines in inducing MDSC with liver damaging potency.

The hallmark of MDSC is their immune suppressive function. Therefore, it is not surprising that various studies provide evidence of MDSC-mediated liver protection [20]–[23]. In these studies, the immune cells causing liver injury were T cells and the degree of liver damage was much more severe than in our study, where MDSC only cause tumor-specific mild liver damage. Naturally, the T cell mediated liver injury could be prevented through MDSC-mediated T cell suppression. Therefore, we argue that the moderate liver damage caused by hepatic MDSC accumulation observed here is ‘collateral damage’, triggered by the same mechanisms that are actually in place to prevent severe forms of liver injury mediated by other immune cells.

## Supporting Information

Figure S1 **Adoptively transferred CD11b^+^ cells accumulate in livers of recipient mice.** 5×10^7^ MACS-sorted hepatic CD45.1^+^CD11b^+^ cells from tumor-bearing mice were injected intravenously into naïve C57BL/6 (CD45.2^+^) mice. Accumulation of transferred cells in the liver of recipient mice was confirmed via detection of CD45.1^+^CD11b^+^Gr-1^+^ cells in the recipient mouse liver via flow cytometry. (N = 2 recipient mice per time point). Data are expressed as mean ±SEM.
(TIFF)

Figure S2 **Cytokine secretion profiles of different tumor models.** Duplicates of tumor-conditioned media (N = 4–6 media samples per tumor cell line culture) were analyzed for interleukin-4, interleukin-10 and interleukin-17 (A). ND  =  not detected. Data are expressed as mean ±SEM.
(TIFF)
